# In Vitro Assessment of Nintedanib in Keratoconus Corneal Stromal Microenvironment

**DOI:** 10.3390/biom16081137

**Published:** 2026-08-05

**Authors:** Yasamin Moradi, Pawan Shrestha, Steve Mabry, Purnima Sharma, Karanpreet S. Multani, Kamran M. Riaz, Dimitrios Karamichos

**Affiliations:** 1 North Texas Eye Research Institute, University of North Texas Health, 3500 Camp Bowie Blvd, Fort Worth, TX 76107, USA; yasaminmoradi@my.unthsc.edu (Y.M.); pawanshrestha@my.unthsc.edu (P.S.); steve.mabry@unthealth.edu (S.M.); purnima04sharma@gmail.com (P.S.); 2 College of Biomedical and Translational Sciences, University of North Texas Health Science Center, 3500 Camp Bowie Blvd, Fort Worth, TX 76107, USA; 3 Department of Family Medicine, Texas College of Osteopathic Medicine, University of North Texas Health Science Center, 3500 Camp Bowie Blvd, Fort Worth, TX 76107, USA; 4 Dean McGee Eye Institute, University of Oklahoma Health Sciences Center, 608 Stanton L. Young Blvd, Oklahoma City, OK 73104, USA; karanpreet-multani@ou.edu (K.S.M.); kamran-riaz@dmei.org (K.M.R.); 5 College of Medicine, University of Oklahoma Health Sciences Center, 800 Stanton L. Young, Oklahoma City, OK 73117, USA

**Keywords:** Nintedanib, keratoconus, cornea, in vitro models, stroma

## Abstract

Keratoconus (KC) is a degenerative corneal disease, characterized by stromal thinning and abnormal ECM remodeling, leading to fibrosis. Corneal fibrosis is a leading cause of visual impairment. Corneal stromal keratocytes differentiate into myofibroblasts, which alters extracellular matrix (ECM) protein deposition. Nintedanib (NIN) is an antifibrotic FDA-approved tyrosine kinase inhibitor, but its function in the cornea is largely unknown. This study examined the impact of NIN within the human corneal stromal microenvironment. Healthy corneal stromal fibroblasts (HCFs) and KC fibroblasts (HKCs) in 2D and 3D in vitro cultures were treated with 1 μM or 2.5 μM NIN. Cell types were evaluated in 2D cultures for metabolic activity, viability, and migration. Protein expression of alpha-smooth muscle actin (α-SMA), collagens (COLs) 1, 3, and 5, cellular fibronectin containing extra domain A (EDA-FN), and thrombospondin-1 (TSP-1) were evaluated in 3D cultures. NIN reduced metabolic activity in HKCs without affecting cell viability. NIN reduced cell migration, downregulated COL3, COL5, EDA-FN, and TSP-1 expression in HCFs and HKCs. COL1 was upregulated in HCFs, whereas α-SMA was upregulated in HKCs. Overall, these findings demonstrate that NIN modulates corneal stromal cell migration and fibrotic marker expression, highlighting its potential as a therapeutic strategy for reducing corneal fibrosis associated with keratoconus.

## 1. Introduction

Keratoconus (KC) is a bilateral and asymmetric disease characterized by progressive corneal ECM thinning and the development of a distinctive cone-like deformity [1,2]. This structural failure causes decreased visual acuity, which results in irregular astigmatism and significant visual distortion [3]. Recent studies suggest that the global prevalence of KC is increasing, with 1.38 per 1000 individuals; however, this number could vary depending on geographic region, along with ethnicity [2,3]. The severity of the disease is typically classified using the Amsler–Krumeich scale, which is frequently applied in clinical practices [4]. While corneal transplantation remains the gold standard for treating severe KC cases, it carries the risk of rejection and long-term complications [2].

Corneal fibrosis can be present in more severe cases of keratoconus [5]. Human corneal fibrosis, or scarring, is characterized by excessive disorganized extracellular matrix (ECM) deposition and is a major cause of visual impairment globally [5,6,7]. Keratocytes, the resident cells of the corneal stroma, are the primary drivers of this pathology. During injury or stress to the cornea, these quiescent keratocytes transform into fibroblasts, which are further activated into myofibroblasts [2,8]. This transformation causes aberrant tissue remodeling and abnormal protein secretion, including collagen types 1, 3, and 5 [9,10,11]. These alterations contribute to corneal fibrosis and possible visual impairment [12].

Corneal fibrosis is challenging to treat, regardless of the cause, due to the complex signaling pathways involved, including transforming growth factor-beta (TGF-β) and connective tissue growth factor (CTGF). These factors are critical for triggering the activation and differentiation of fibroblasts into myofibroblasts [13,14,15,16,17], excessive ECM deposition, and tissue remodeling [18,19]. The inhibition of these pathways is known to play a role in reducing fibrotic phenotypes both in in vitro and in vivo models [20,21,22]. Nintedanib (NIN) is an FDA-approved antifibrotic drug, originally developed for treating idiopathic pulmonary fibrosis (IPF), a chronic lung disease characterized by excessive fibrosis [23]. NIN is a tyrosine kinase inhibitor of multiple receptors that binds competitively to the adenosine-5′-triphosphate (ATP) binding site [24], including the vascular endothelial growth factor receptor (VEGFR), platelet-derived growth factor receptor (PDGFR), and fibroblast growth factor receptor (FGFR) [23,25,26,27]. Studies using human lung fibroblasts and primary human and rat lung tissue, as well as human dermal fibroblast models, have demonstrated that inhibiting these receptors and their downstream pathways significantly modulated and downregulated ECM proteins, such as fibronectin and collagens 1, 3, and 5, thereby reducing fibrotic activity and improving tissue function [28,29,30]. Studies in preclinical models of systemic sclerosis, bleomycin-induced pulmonary fibrosis, and tracheal stenosis have shown that NIN inhibits profibrotic cytokines such as CTGF and TGF-β1, thereby suppressing fibroblast activation, proliferation, migration, and ECM deposition [26,28,31,32,33,34,35]. NIN’s anti-neovascularization effects have also been reported in ocular injury models, where topical NIN eye drops significantly reduced corneal neovascularization (NV) in alkali-burned rat and rabbit corneas, suggesting its ability to inhibit pro-angiogenic signaling in the cornea [36,37,38]. Effective ocular drug delivery for corneal NV remains a significant challenge, with more recent nanocarrier-based studies aiming at enhancing drug penetration and therapeutic efficacy [39,40,41]. Recently, NIN has demonstrated anti-scarring and therapeutic effects in several ocular models, including corneal injury, extraocular muscle surgery, and glaucoma filtration surgery [42,43,44]. However, studies on NIN in the human cornea remain extremely limited. Given the shared fibrotic pathophysiology between lung and corneal tissues, such as excessive ECM deposition and TGF-β-driven myofibroblast activation [45,46], this study aimed to evaluate the effect of NIN on the corneal stroma and KC microenvironment. To our knowledge, this study is the first of its kind, paving the way for future discoveries.

## 2. Materials and Methods

### 2.1. Ethics Approval

This study adhered to the tenets of the Declaration of Helsinki. Human cadaver corneas without any history of ocular or systemic disease were obtained from the National Disease Research Interchange (NDRI, Philadelphia, PA, USA). Human corneas with a history of KC were recovered from Dean McGee Eye Institute (Oklahoma City, OK, USA) with Institutional Research Board (IRB) approval (IRB# 10108). All corneas used within the study were de-identified prior to use and were approved by the North Texas Regional Institutional Review Board (IRB # 2020-030).

### 2.2. Isolation of Corneal Stromal Cells

Human corneal stromal cells were isolated from healthy (HCFs) and KC (HKCs) donor corneas, as previously described [9,47]. Cells were cultured in complete medium: EMEM (EMEM; CORNING, Corning, NY, USA) containing 10% FBS (FBS: GeminiBio Inc., West Sacramento, CA, USA) and 1% antibiotic–antimycotic (A.A.; Gibco, Life Technologies; Grand Island, NY, USA) in a humidified incubator at 37 °C with 5% CO_2_. Cell passages 5-6 were used throughout the experiments. All HCFs and HKCs used in these experiments originated from the same healthy and KC donors, respectively.

### 2.3. Metabolic Activity Assay

Cellular metabolic activity was measured using the MTT Assay (Invitrogen, Thermo Scientific, Waltham, MA, USA). A total of 5 × 10^4^ cells/well were seeded in a 96-well culture plate (Thermo Scientific, BioLite, Waltham, MA, USA) with complete medium. The next day, HCFs and HKCs were treated with either 1 μM or 2.5 μM NIN, while complete medium was used as the control. After 24 h, 10 μL of the 12 mM MTT stock solution was added to each well, followed by incubating the plates for 3 h at 37 °C in a humidified atmosphere containing 5% CO_2_. After incubation, 50 μL of DMSO was added, and the mixture was incubated for 10 min at 37 °C. Absorbance was measured at 540 nm using a spectrometer (Epoch2; BioTek, Winooski, VT, USA). The results were analyzed in Excel and GraphPad Prism version 10.4.1.

### 2.4. Cell Viability

Live/Dead Viability/Cytotoxicity Assay for Mammalian Cells (Invitrogen, Thermo Scientific, Waltham, MA, USA) containing Calcein AM and ethidium homodimer-1 (EthD-1) was used to assess cell viability. HCFs and HKCs (2.5 × 10^5^) were seeded in a 12-well plate with complete medium and allowed to attach overnight, followed by treatment with either 1 μM or 2.5 μM NIN. The next day, the medium was removed and wells were washed with Dulbecco’s phosphate-buffered saline (DPBS; Thermo Scientific, Waltham, MA, USA). The staining solution was prepared according to the manufacturer’s instructions and added to each well. The plates were incubated for 30 min at room temperature, protected from light. Live cells were identified by green Calcein AM fluorescence at excitation and emission wavelengths of 485 and 530 nm, and dead cells were identified by red EthD-1 fluorescence at excitation and emission wavelengths of 530 and 645 nm, using a Synergy LX microplate reader (BioTek, Winooski, VT, USA). Representative fluorescent images were taken by an automated fluorescence imaging system (Cytation 5 Cell Imaging Multi-Mode Reader; BioTek, Winooski, VT, USA).

### 2.5. Cell Migration Assay

To assess cell migration, a 2D in vitro scratch assay was employed. HCFs and HKCs were seeded at a density of 1 × 10^6^ cells/well on flat 6-well culture plates without pre-coating the wells with additional matrix protein (Thermo Scientific, BioLite, Waltham, MA, USA). Both HCFs and HKCs were allowed to adhere for 24 h in complete medium. After 24 h, the medium was replaced: the control group received fresh complete medium, whereas the treatment groups were supplemented with 1 μM or 2.5 μM NIN. Using a 10 µL pipette tip, a scratch was made through the confluent cell layer. Images of the scratches were taken at 0, 6, 12, 24, 48, and 72 h using an AmScope IN200TA-M Digital Long Working Distance Inverted Trinocular Microscope (AmScope, Irvine, CA, USA) to track wound closure. Cell migration was quantified by analyzing the images using ImageJ software version 1.54g.

### 2.6. Assembly of 3D Constructs

Both HCFs and HKCs were seeded at a density of 1 × 10^6^ cells per well onto polycarbonate inserts with 0.4 μm pores in 6-well transwell plates (Transwell; Corning Costar, Charlotte, NC, USA) as previously described [9,48,49]. Control HCF and HKC groups were cultured with complete medium supplemented with 0.5 mM stable Vitamin C (0.5 mM 2-O-α-D-glucopyranosyl-L-ascorbic acid, Sigma-Aldrich, St. Louis, MO, USA) to stimulate ECM secretion and assembly. NIN-treated groups were cultured with complete medium, Vitamin C, and the addition of 1 μM or 2.5 μM of NIN. NIN (Nintedanib; Tocris Bioscience, Avonmouth, Bristol, UK) was dissolved in dimethyl sulfoxide (DMSO) and prepared at a stock concentration of 1 mM. All cultures were maintained for four weeks, with medium changes every other day.

### 2.7. Protein Extraction and Quantification

After four weeks, all 3D constructs were washed three times with DPBS and removed from the membranes. A total of 100 µL of Cell Lysis Buffer II (CLBII; Invitrogen, Waltham, MA, USA) supplemented with 10% protease inhibitor cocktail (P.I.; Sigma Aldrich, St. Louis, MO, USA) was added to each well and incubated at 4 °C overnight. The cell supernatant containing the proteins was collected by centrifugation at 12000 RPM for 15 min at 4 °C. Protein concentrations were determined using the Pierce™ BCA Protein Assay Kit (Thermo Scientific, Waltham, MA, USA), based on the manufacturer’s instructions. A series of bovine serum albumin (BSA) standards (Thermo Scientific, Waltham, MA, USA) were prepared for the assay. For quantification, 10 µL of each protein sample and standard was added to a 96-well plate (Corning Costar, Thermo Scientific), followed by placing the plate on a shaker for 30 s. The plate was then incubated for 30 min at 37 °C with 5% CO_2_. Absorbance was measured at 562 nm using a BioTek EPOCH2 microplate reader (BioTek, Winooski, VT, USA). Protein concentrations were calculated by plotting the absorbance values against the standard curve generated from the BCA standards (Bovine Gamma Globulin Standard Pre-Diluted Set, PI23213; Thermo Scientific, Waltham, MA, USA) using linear regression.

### 2.8. Western Blot Analysis

Protein samples were loaded onto Novex 4–20% Tris-glycine mini wedge 10-well gels (Invitrogen, Thermo Scientific, Waltham, MA, USA) and were run at 225 V for 1 h and 30 min. After electrophoresis, the proteins were transferred onto PDVF membranes using the iBlot 2 Dry Blotting System (Invitrogen, Thermo Scientific, Waltham, MA, USA). The membranes were blocked with 1× Blocker™ FL fluorescent blocking buffer (Thermo Scientific, Waltham, MA, USA) at room temperature for 1 h on a rocker. Following blocking, the membranes were incubated overnight at 4 °C with primary antibodies diluted 1:500 including α-SMA (ab5694; Abcam, Cambridge, MA, USA), collagen 3 (COL3; ab184993; Abcam, Cambridge, MA, USA), EDA-FN (SAB4200784; Millipore Sigma, Burlington, MA, USA), COL1 (ab138492; Abcam, Cambridge, MA, USA), COL5 (ab275881; Abcam, Cambridge, MA, USA), TSP-1 (ab85762; Abcam, Cambridge, MA, USA), and β-actin (ab184092; Abcam, Cambridge, MA, USA). The next day, the membranes were washed three times, each for 5 min with Tris-buffered saline containing 0.1% Tween 20 (TBST). Secondary antibodies were applied and incubated for 1 h at room temperature. After additional washing with TBST, the membranes were imaged using the iBright FL 15,000 imaging system (Thermo Scientific, Waltham, MA, USA). Image analysis was performed using iBright analysis software version 5.5.0 (Thermo Scientific, Waltham, MA, USA). All protein expressions were normalized to β-actin (housekeeping protein) and the results were plotted.

### 2.9. Statistical Analysis

All experiments were conducted with a minimum of three replicates (*n* = 3). Statistical significance was determined using two-way ANOVAs for comparisons between disease (HCF versus HKC) and NIN treatment conditions (Control, 1 μM, 2.5 μM). For comparisons within either HCF or HKC measuring the effects of NIN treatment conditions, one-way ANOVAs were performed. All analyses were followed by Fisher’s least significant difference (LSD) post hoc test. GraphPad Prism version 10.4.1 was utilized for both statistical analysis and graphical representation. Data are presented as the mean ± S.E.M. Statistical significance between treatment groups and their respective controls is indicated by asterisks (* *p* < 0.05, ** *p* < 0.01, *** *p* < 0.001, **** *p* < 0.0001), while statistical significance between HCF and HKC groups is indicated by dollar signs ($ *p* < 0.05, $$ *p* < 0.01, $$$ *p* < 0.001, $$$$ *p* < 0.0001). A *p* value < 0.05 was considered statistically significant.

## 3. Results

### 3.1. Impact of NIN on Cell Viability and Metabolic Activity in HCFs and HKCs

**Figure 1:**
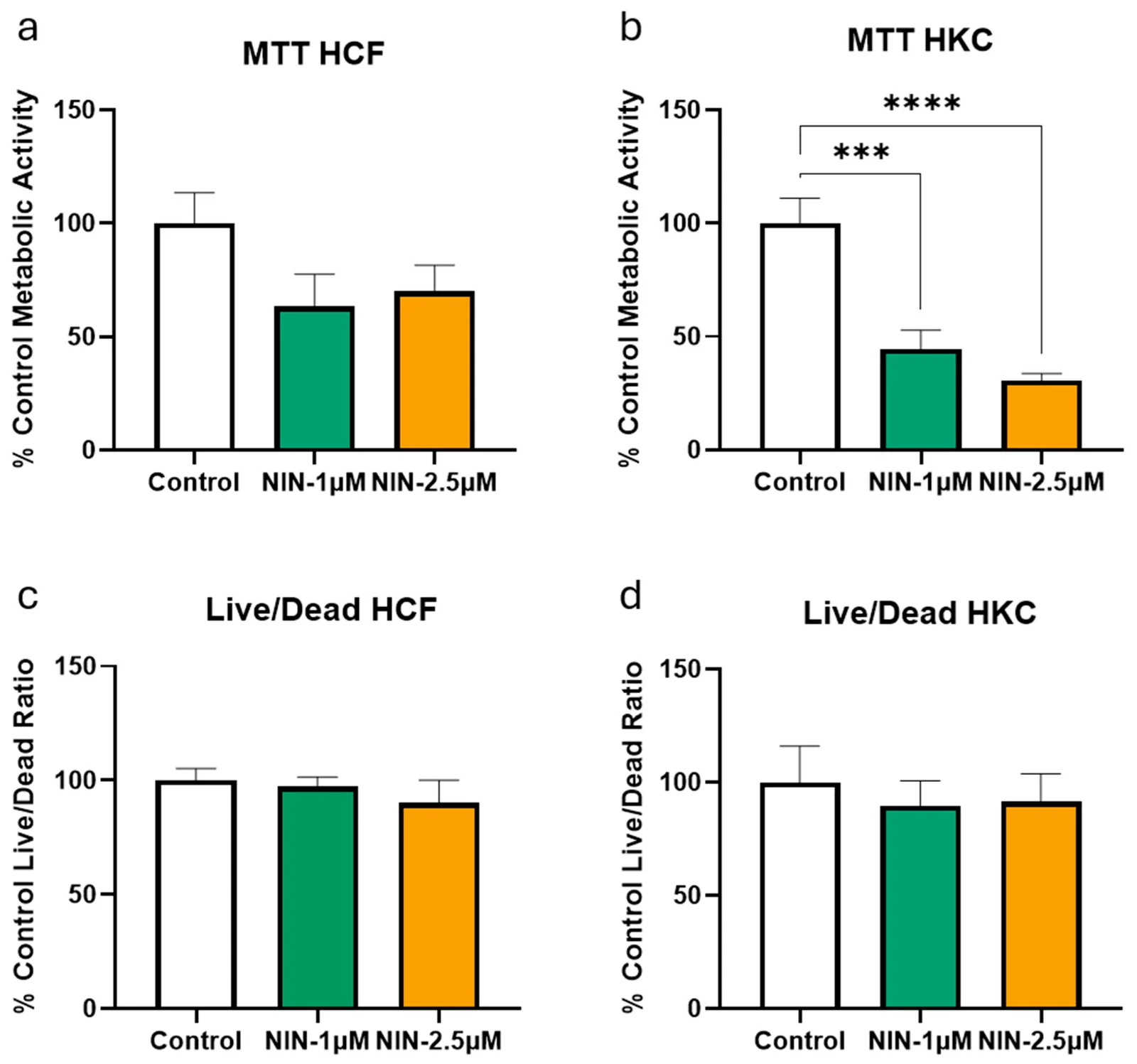
(**a**) MTT assay showing the percentage of cellular metabolic activity in HCFs treated with NIN (1 μM and 2.5 μM) compared to control cells. (**b**) MTT assay showing the percentage of cellular metabolic activity in HKCs treated with NIN (1 μM and 2.5 μM) compared to control cells. (**c**) Live/dead assay showing the relative live/dead fluorescence ratio in HCFs treated with NIN (1 μM and 2.5 μM), normalized to the untreated control. (**d**) Live/dead assay showing the relative live/dead fluorescence ratio in HKCs treated with NIN (1 μM and 2.5 μM), normalized to the untreated control. Data are presented as mean ± S.E.M. (*n* = 3). There is no significant difference between data unless otherwise noted with the following symbol: *** *p* < 0.001, **** *p* < 0.0001.

The MTT assay showed that NIN did not have a significant impact on the metabolic activity of HCFs at either concentration (Figure 1a). In contrast, NIN treatment significantly reduced the metabolic activity of HKCs at both 1 μM and 2.5 μM compared with the control (*p* = 0.0001; Figure 1b). Results from the live/dead assay (Figure 1c,d), with the relative live/dead ratio normalized to the untreated control, demonstrated that NIN did not have a significant effect on the live/dead ratio in either HCFs or HKCs at either concentration.

### 3.2. NIN Inhibits Cell Migration in HCF and HKC

**Figure 2:**
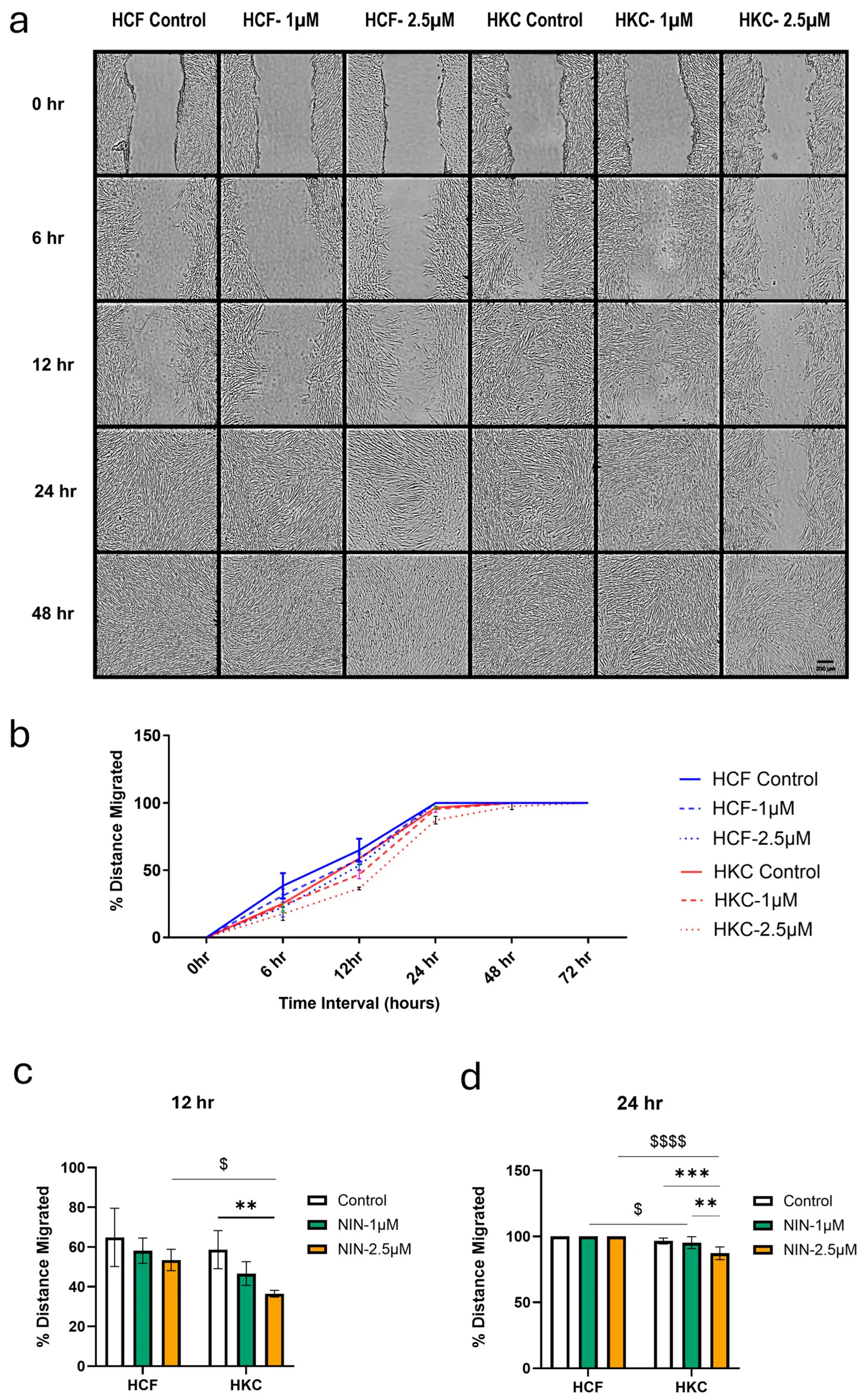
(**a**) Representative microscope images of the scratch assay at 0, 2, 6, 12, 24, and 48 h post-scratching for HCF and HKC cells treated with NIN (1 μM and 2.5 μM). The scale bar represents 200 μm. (**b**) Quantification of the percentage of total cell migration distance over time for HKC and HCF cells treated with NIN (1 μM and 2.5 μM) at 0, 6, 12, 24, 48, and 72 h (*n* = 3). (**c**,**d**) Statistical analysis of migration assay at 12 h (**c**) and 24 h (**d**). Data are presented as mean ± S.E.M. There is no significant difference between data unless otherwise noted with the following symbols: ** *p* < 0.01, *** *p* < 0.001, $ *p* < 0.05, $$$$ *p* < 0.0001.

Scratch assay was conducted to determine the effect of NIN on cell migration for both healthy human corneal stromal fibroblasts (HCFs) and KC fibroblasts (HKCs) (Figure 2a). There were no significant differences in cell migration at 6 h (Figure 2b). At 12 h, 2.5 μM NIN inhibited cell migration in HKCs compared to control HKCs (*p* = 0.0034) and 2.5 μM NIN-treated HCFs (*p* = 0.0244; Figure 2c). At 24 h, all HCF scratches were closed (Figure 2b). In HKCs at 24 h, we observed that 2.5 μM NIN decreased cell migration compared to controls (*p* = 0.0144) and compared to 1 μM NIN (*p* = 0.0274; Figure 2d). Only 2.5 μM NIN-treated HKCs were not closed by 48 h, and all scratches were closed by 72 h (Figure 2b).

### 3.3. NIN Downregulates ECM Markers

**Figure 3:**
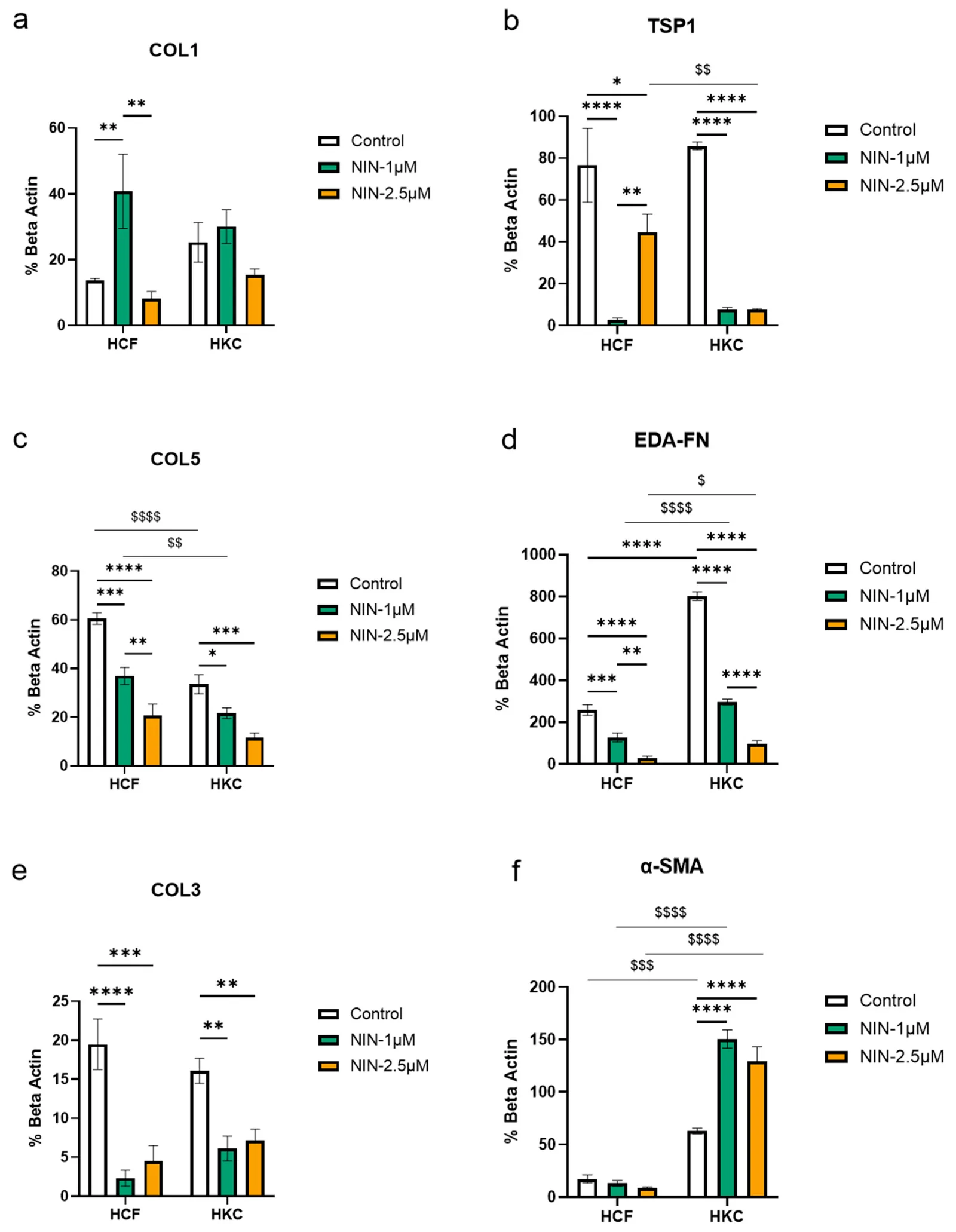
Expression levels of (**a**) COL1, (**b**) TSP-1, (**c**) COL5, (**d**) EDA-FN, (**e**) COL3, and (**f**) α-SMA in HCF and HKC cells treated with either 1 μM NIN or 2.5 μM NIN compared to untreated control (*n* = 3). All data represent protein expression as a percent of housekeeping protein (Beta Actin) control. Data is presented as mean ± S.E.M. There are no significant differences between bars unless otherwise indicated. Statistical significance between treatment groups and their respective controls is indicated by asterisks (* *p* < 0.05, ** *p* < 0.01, *** *p* < 0.001, **** *p* < 0.0001). Statistical significance between HCF and HKC groups is indicated by dollar signs ($ *p* < 0.05, $$ *p* < 0.01, $$$ *p* < 0.001, $$$$ *p* < 0.0001). Original Western Blots images for Figure 3 are found in Supplementary Materials.

Protein expression levels of alpha-smooth muscle actin (α-SMA), COL3, cellular fibronectin containing extra domain A (EDA-FN), COL1, COL5, and TSP-1 were analyzed across all 3D cultures and cell types (Figure 3a–f). In HCFs, treatment with 1 µM NIN led to a significant increase in COL1 compared to the controls (*p* = 0.0061) and 2.5 μM NIN (*p* = 0.0017; Figure 3a). No changes were observed with 2.5 µM NIN treatment (Figure 3a). In HKCs, treatment with NIN (1 µM and 2.5 µM) showed no significant changes in COL1 expression at either concentration (Figure 2a).

TSP-1 expression was markedly downregulated in HCFs, in both 1 µM (*p* < 0.0001) and 2.5 µM (*p* = 0.0159) NIN treatments (Figure 3b). However, TSP-1 expression in HCFs was greater at 2.5 μM NIN than at 1 μM NIN (*p* = 0.0033; Figure 3b). TSP-1 expression was significantly downregulated at both concentrations in HKCs (*p* < 0.0001), with no difference between 1 and 2.5 μM NIN (Figure 3b). TSP-1 expression at 2.5 μM NIN was higher in HCFs than in HKCs at the same dose (Figure 3b).

COL5 was significantly downregulated at both NIN concentrations in HCFs (*p* = 0.0002 for 1 µM; *p* < 0.0001 for 2.5 µM, Figure 3c). Overall, 2.5 μM NIN-treated HCFs had greater downregulation of COL5 than 1 μM NIN (*p* = 0.0040; Figure 3c). We observed a similar pattern in downregulation of COL5 in HKCs at both 1 μM (*p* = 0.0232) and 2.5 µM NIN (*p* = 0.0005), though there was no difference between NIN treatments (Figure 3c). COL5 expression was greater in HCF controls (*p* < 0.0001) and 1 μM NIN-treated HCFs (*p* = 0.0058) compared to HKCs of the same conditions (Figure 3c).

In HCFs, EDA-FN was downregulated at 1 µM (*p* = 0.0003) and 2.5 µM (*p* < 0.0001), though 1 μM NIN was less downregulated than 2.5 μM (*p* = 0.0031; Figure 3d). This effect of NIN was mirrored in HKCs, with 1 μM (*p* < 0.0001) and 2.5 μM NIN (*p* < 0.0001) downregulated compared to controls, and greater downregulation observed in 2.5 μM NIN compared to 1 μM NIN (*p* < 0.0001; Figure 3d). In controls (*p* < 0.0001) and both NIN doses (*p* < 0.0001 for 1 µM; *p* = 0.0301 for 2.5 µM), HKCs had greater expression of EDA-FN than HCFs (Figure 3d). Much like EDA-FN, COL3 expression in HCFs was significantly downregulated at both 1 µM (*p* < 0.0001) and 2.5 µM (*p* = 0.0002; Figure 3e). We observed a similar pattern of downregulation in HKC COL3 expression, which was significantly downregulated at 1 µM (*p* = 0.0035) and 2.5 µM (*p* = 0.0070; Figure 3e). There was no difference in COL3 expression between HKCs and HCFs (Figure 3e).

In HCFs, expression of α-SMA remained unchanged across both NIN concentrations (Figure 3f). Conversely, NIN upregulated α-SMA expression in HKCs at both concentrations (*p* < 0.0001 for 1 µM; *p* < 0.0001 for 2.5 µM) compared with the control (Figure 3f). α-SMA expression was greater in HKC controls (*p* = 0.0007), 1 μM NIN-treated HKCs (*p* < 0.0001), and 2.5 μM NIN-treated HKCs (*p* < 0.0001) compared to HCFs of the same conditions (Figure 3f).

## 4. Discussion

Studies showed NIN’s role in improving outcomes for patients affected by lung fibrosis by reducing the risk of all-cause mortality and acute exacerbations across both IPF and non-IPF fibrosing interstitial lung diseases (ILDs) [23,35,50]. NIN not only inhibits fibroblast proliferation and activation but also exhibits anti-inflammatory properties, including its activity against various cytokine pathways [28,32,51,52]. In TGF-β and PDGF-stimulated cardiac and pulmonary fibroblasts, NIN was shown to reduce pSTAT3 levels, an effect correlated with pro-apoptotic ER stress, a pathway linked to inflammation [53]. For KC, several drug candidates are being explored to target the underlying mechanisms of corneal fibrosis [1]. Riboflavin, commonly used in corneal collagen cross-linking, may indirectly influence corneal fibrosis by stabilizing stromal architecture and reducing mechanical strain, which in turn can reduce TGF-β-driven myofibroblast activation and extracellular-matrix remodeling [54,55]. Quercetin, an endogenous compound with strong antioxidant and anti-inflammatory properties, has been investigated for its potential to modulate cellular metabolism in the corneal stroma and stabilize keratocyte function in keratoconus [56,57,58]. TGF-β inhibitors, which directly target the underlying signaling pathways involved in myofibroblast activation and ECM deposition, are also being explored as a potential treatment to prevent or reverse the fibrotic ECM remodeling in the cornea and KC [12,59,60,61]. The ongoing research into non-surgical treatments for KC highlights the importance of finding strategies to preserve corneal function and reduce the need for corneal transplantation [62].

Early KC is characterized by biomechanical weakening of the corneal stroma with minimal fibrotic involvement, whereas advanced disease reflects a shift towards stromal wound-healing responses and activation of fibrotic signaling pathways [1,2,8,61]. This transition highlights fibrosis-related mechanisms as potential therapeutic targets and supports the investigation of antifibrotic agents such as NIN in advanced KC [54,55,63,64]. Although NIN has not been directly studied in KC, its ability to inhibit key signaling pathways involved in fibrosis makes it a promising candidate for investigation in this condition [54,55,65,66,67]. Clinical trials, including INPULSIS and TOMORROW trials [35,68,69], provided evidence that NIN significantly reduced the decline in forced vital capacity (FVC), which is a standard measure of lung function, compared to placebo [70].

Results from this study showed that although NIN significantly reduced metabolic activity of HKCs, the live/dead assay demonstrated no significant changes in cell viability in either HKCs or HCFs. This suggests the decrease in metabolic activity is not associated with increased cell death, indicating that NIN modulates cell behavior rather than causing cytotoxicity [71,72,73].

We observed that only 2.5 μM NIN inhibited cell migration in HKCs compared to controls, with no effect of 1 μM NIN in HKCs, or either dose of NIN observed in HCFs. This is consistent with previous studies on NIN’s role in limiting the migratory activity of keloid fibroblasts, peritoneal mesothelial cells, and lung fibroblasts [74,75,76]. Fibroblast migration is a key cellular process driving fibrotic changes in the cornea [77,78]. By limiting cell movement, NIN may prevent further ECM accumulation, which is a KC hallmark [28,46,79].

The protein analysis revealed significant changes in the expression of key fibrotic markers in response to NIN treatment, including COL3, EDA-FN, COL5, and TSP1, in HCFs and HKCs. These findings align with previous studies in non-corneal tissues such as the lungs, liver, and skin, showing that NIN decreases ECM components [28,80,81,82]. However, in contrast to these models, NIN treatment in our study resulted in a simultaneous increase in α-SMA and COL1 expression in HCFs and HKCs which can indicate that, although NIN reduces the accumulation of ECM components such as COL3, its impact on myofibroblast activity may differ. The selective increase in COL1 expression observed in HCFs further suggests that healthy and keratoconus-derived corneal fibroblasts may exhibit distinct responses to NIN. Although the present study was not designed to investigate the underlying molecular mechanisms, future studies examining upstream regulatory pathways, including TGF-β signaling, may help explain these cell-specific responses. Prior studies in lung fibroblasts and fibrocytes have shown that NIN suppresses ECM protein secretion, inhibits myofibroblast differentiation and reduces α-SMA expression [28,39,76,83].

The NIN concentrations tested in this study were selected following an initial in vitro concentration series designed to determine the optimal range for the drug in our 3D model [82,84]. The initial concentrations tested were 1 μM, 2.5 μM, and 5 μM. We observed significant ECM contraction at 5 μM NIN, whereas lower concentrations (1 μM and 2.5 μM) supported stable ECM assembly. Therefore, 5 μM and higher concentrations were not investigated further in this study. Notably, the lower concentrations of NIN showed significant changes in protein expression, while the higher concentration induced contractile effects. These findings highlight a study limitation: higher NIN concentrations are unsuitable for our 3D model due to their impact on ECM integrity. While our 3D system remains a physiologically relevant platform compared to 2D cultures, further studies are needed to optimize NIN dosing to achieve maximal antifibrotic benefits without compromising ECM stability in HCFs and HKCs. Furthermore, the current studies do not explore biomechanical responses or potential donor-dependent variabilities. Although this proof-of-concept study provides initial evidence of the antifibrotic effects of NIN, future studies incorporating multiple independent donor-derived cells will be important to validate these findings. Future studies will also determine whether the observed findings in HKCs reflect a compensatory or NIN-driven response. These gaps present opportunities for future studies to enhance therapeutic translation.

## 5. Conclusions

In conclusion, this study demonstrates that NIN may be a potential therapeutic agent for KC, particularly by influencing the fibrotic changes associated with disease progression. NIN targets the molecular pathways involved in fibrosis, reducing ECM accumulation, inhibiting myofibroblast activation, and ultimately preserving corneal function in KC. However, the observed differential effects in HCFs and HKCs suggest that further research is needed to better understand the cell-specific mechanisms and the underlying cellular microenvironment factors and pathways through which NIN may exhibit antifibrotic effects.

## Data Availability

The data presented in this study are openly available in [Figshare] [10.6084/m9.figshare.32305248] https://figshare.com/s/26f9dd1bbd6e11e70f91, accessed on 24 July 2026.

## Supplementary Materials

The following supporting information can be downloaded at: https://www.mdpi.com/article/10.3390/biom16081137/s1, Figure S1: Western Blot images and Total Density values of β-Actin protein expression in HCF and HKC 3D constructs following NIN treatment after 4 weeks. Constructs without treatment serve as controls. Each condition was repeated 3 times; Figure S2. Western Blot images and Total Density values of COL5 protein expression in HCF and HKC 3D constructs following NIN treatment after 4 weeks. Constructs without treatment serve as controls. Each condition was repeated 3 times; Figure S3. Western Blot images and Total Density values of β-Actin protein expression in HCF and HKC 3D constructs following NIN treatment after 4 weeks. Constructs without treatment serve as controls. Each condition was repeated 3 times; Figure S4. Western Blot images and Total Density values of EDA-FN protein expression in HCF and HKC 3D constructs following NIN treatment after 4 weeks. Constructs without treatment serve as controls. Each condition was repeated 3 times; Figure S5. Western Blot images and Total Density values of Tsp1 protein expression in HCF and HKC 3D constructs following NIN treatment after 4 weeks. Constructs without treatment serve as controls. Each condition was repeated 3 times; Figure S6. Western Blot images and Total Density values of COL3 protein expression in HCF and HKC 3D constructs following NIN treatment after 4 weeks. Constructs without treatment serve as controls. Each condition was repeated 3 times; Figure S7. Western Blot images and Total Density values of COL1 protein expression in HCF and HKC 3D constructs following NIN treatment after 4 weeks. Constructs without treatment serve as controls. Each condition was repeated 3 times; Figure S8. Western Blot images and Total Density values of α-SMA protein expression in HCF and HKC 3D constructs following NIN treatment after 4 weeks. Constructs without treatment serve as controls. Each condition was repeated 3 times.
