# Supplementary material for: In Vitro Assessment of Nintedanib in Keratoconus Corneal Stromal Microenvironment
Source: Biomolecules. 2026 Aug 5;16(8):1137. doi: 10.3390/biom16081137 (PMC13511102; doi:10.3390/biom16081137)
Supplement: Supplementary file 1 [file biomolecules-16-01137-s001.zip › biomolecules-4351075-supplementary.pdf]

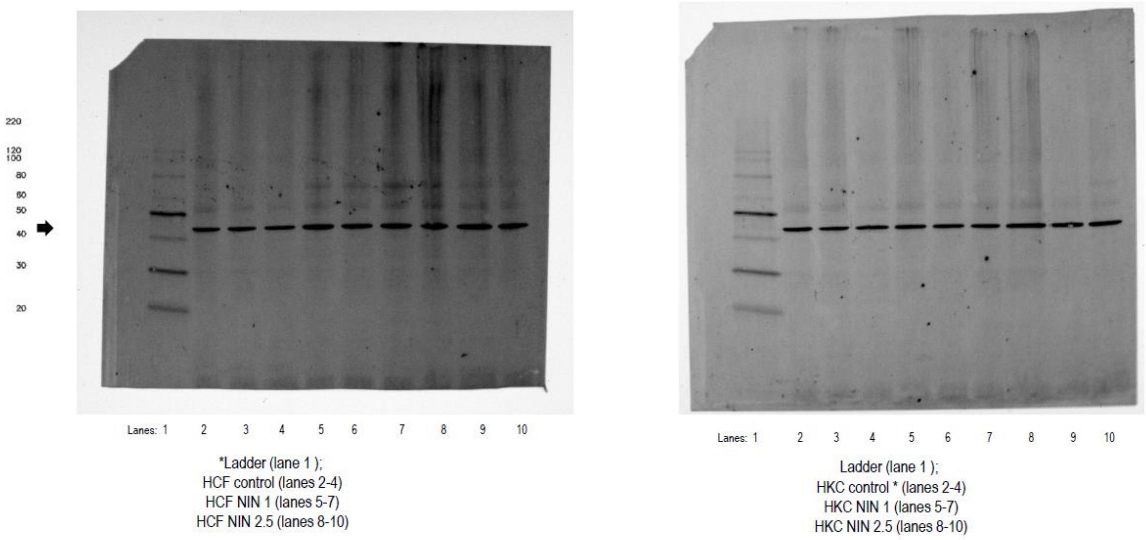

Figure S1. Western Blot images and Total Density values of β-Actin protein expression in HCF and HKC 3D constructs following NIN treatment after 4 weeks. Constructs without treatment serve as controls. Each condition was repeated 3 times.

COL5 molecular weight- 180 kDa; **MagicMark ladder molecular weights** = 20, 30, 40, 50, 60, 80, 100, 120, and 220 kDa

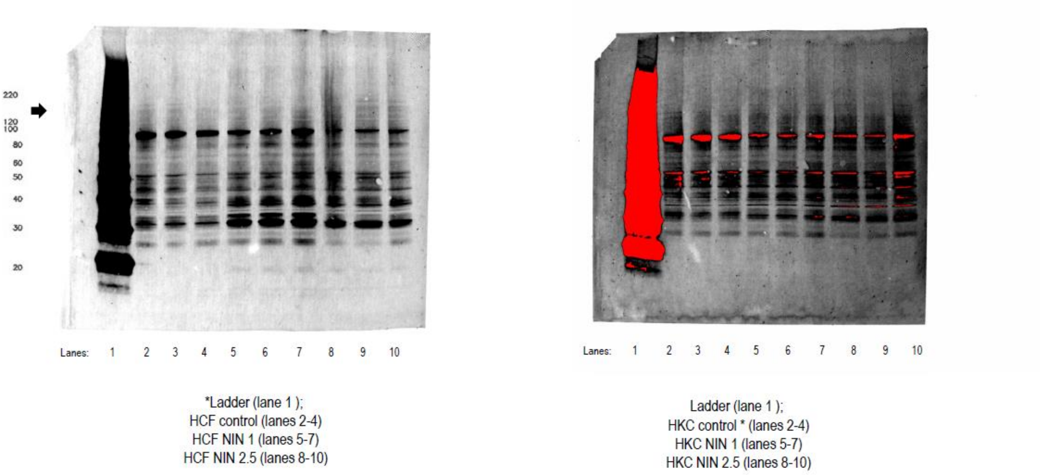

Figure S2. Western Blot images and Total Density values of COL5 protein expression in HCF and HKC 3D constructs following NIN treatment after 4 weeks. Constructs without treatment serve as controls. Each condition was repeated 3 times.

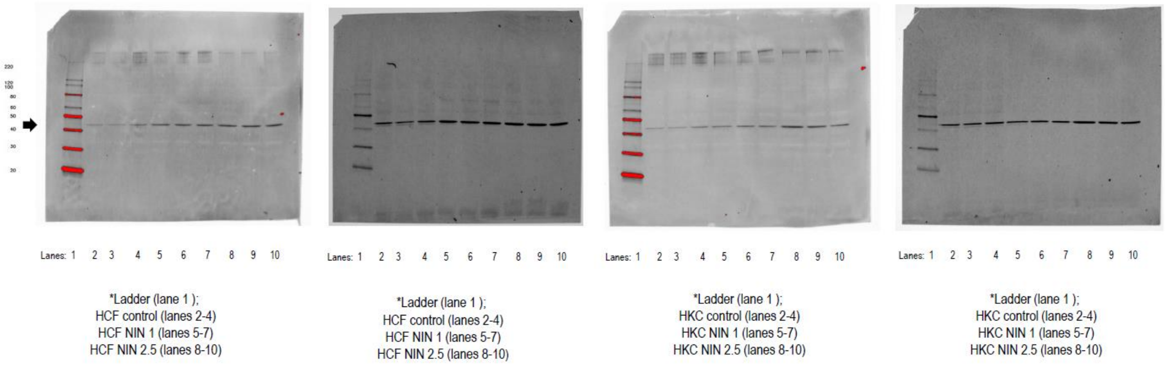

Figure S3. Western Blot images and Total Density values of β-Actin protein expression in HCF and HKC 3D constructs following NIN treatment after 4 weeks. Constructs without treatment serve as controls. Each condition was repeated 3 times.

EDA-FN molecular weight- 220 kDa; **MagicMark ladder molecular weights** = 20, 30, 40, 50, 60, 80, 100, 120, and 220 kDa

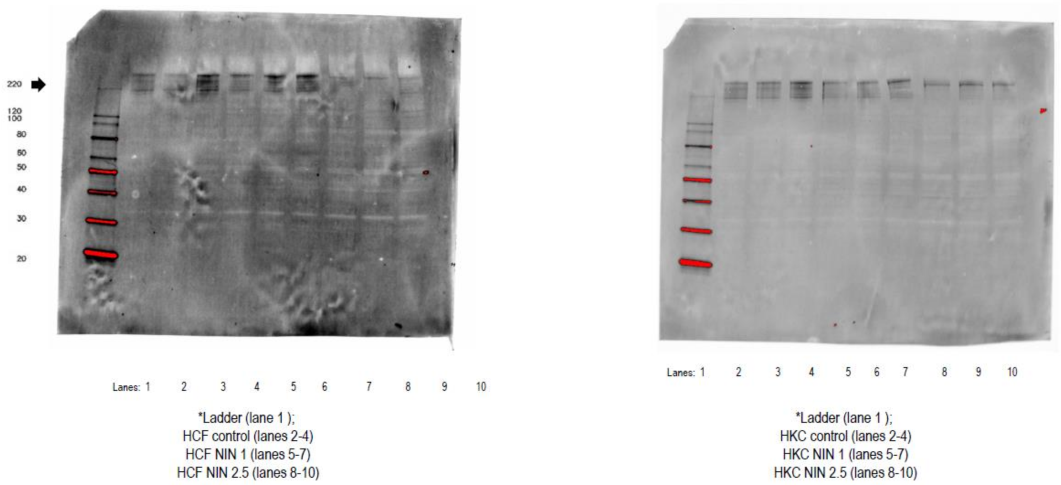

Figure S4. Western Blot images and Total Density values of EDA-FN protein expression in HCF and HKC 3D constructs following NIN treatment after 4 weeks. Constructs without treatment serve as controls. Each condition was repeated 3 times.

Tsp1 molecular weight- 130 kDa; **MagicMark ladder molecular weights** = 20, 30, 40, 50, 60, 80, 100, 120, and 220 kDa

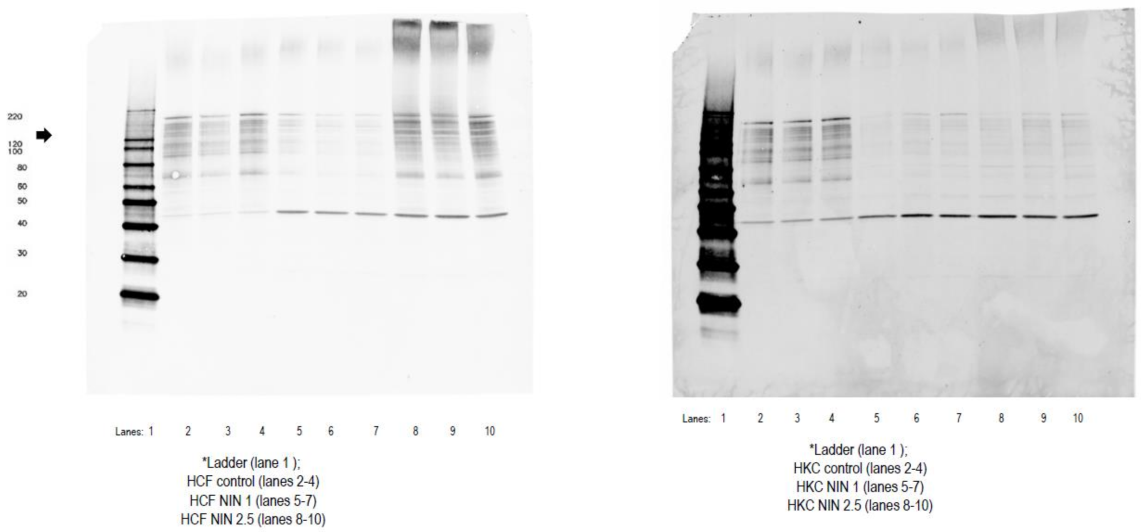

Figure S5. Western Blot images and Total Density values of Tsp1 protein expression in HCF and HKC 3D constructs following NIN treatment after 4 weeks. Constructs without treatment serve as controls. Each condition was repeated 3 times.

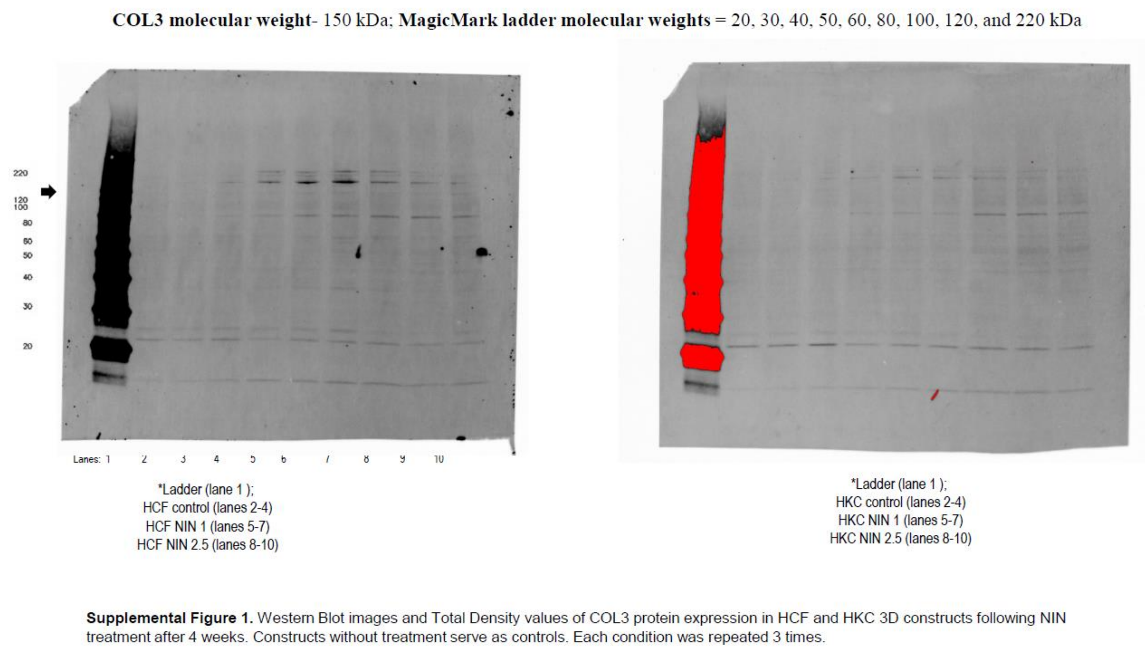

Figure S6. Western Blot images and Total Density values of COL3 protein expression in HCF and HKC 3D constructs following NIN treatment after 4 weeks. Constructs without treatment serve as controls. Each condition was repeated 3 times.

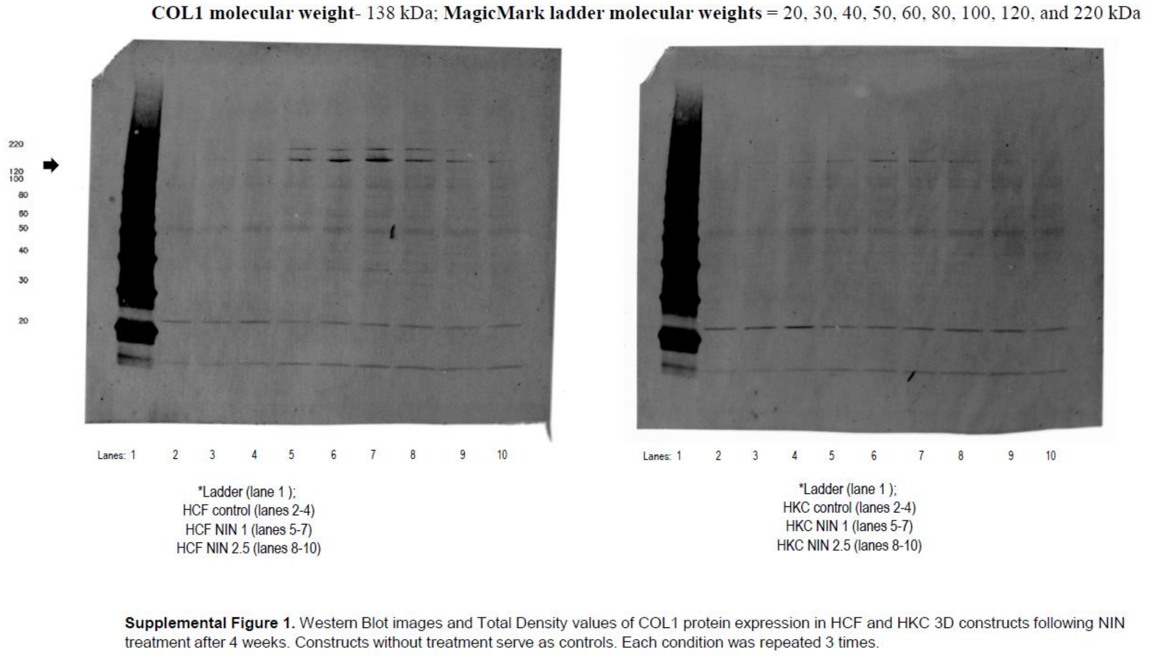

Figure S7. Western Blot images and Total Density values of COL1 protein expression in HCF and HKC 3D constructs following NIN treatment after 4 weeks. Constructs without treatment serve as controls. Each condition was repeated 3 times.

α-SMA molecular weight- 42 kDa; **MagicMark ladder molecular weights** = 20, 30, 40, 50, 60, 80, 100, 120, and 220 kDa

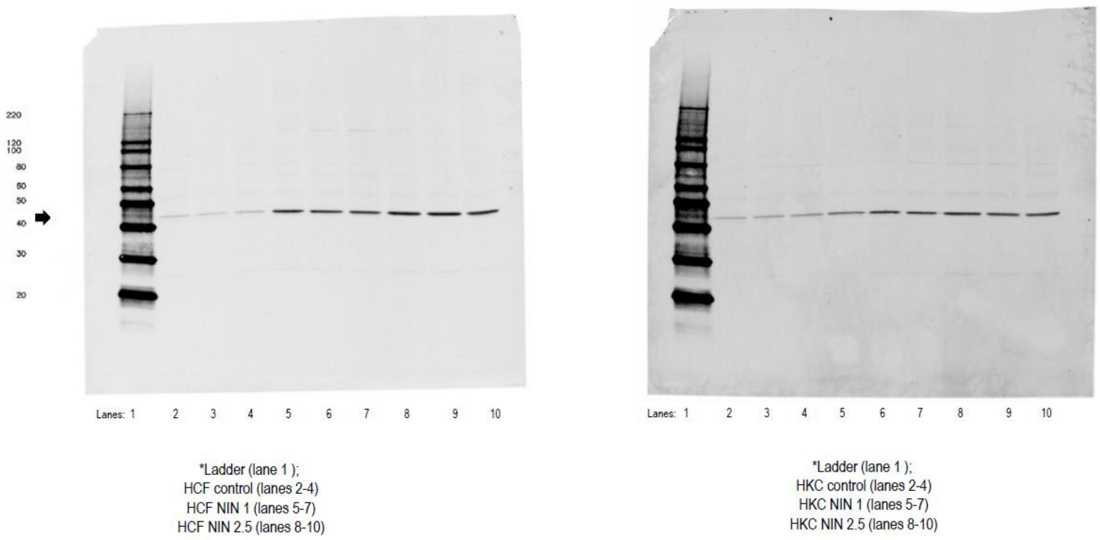

Figure S8. Western Blot images and Total Density values of α-SMA protein expression in HCF and HKC 3D constructs following NIN treatment after 4 weeks. Constructs without treatment serve as controls. Each condition was repeated 3 times.
